# The test-retest reliability of cortical activation and behavioral metrics in adolescent basketball players during dual-task postural control: an fNIRS-based assessment

**DOI:** 10.3389/fpsyg.2026.1948562

**Published:** 2026-10-09

**Authors:** Wenyi Li, Junye Tao, Gede Zheng, Jun Chen, Lulu Song

**Affiliations:** 1 Department of Sports, University of Electronic Science and Technology of China, Chengdu, Sichuan, China; 2 College of Physical Education and Sports, Beijing Normal University, Beijing, China; 3 Capital University of Physical Education and Sports, Beijing, China

**Keywords:** adolescent athletes, cortical activation, dual-task, functional near-infrared spectroscopy, postural control, test-retest reliability

## Abstract

**Background:**

Athletic performance in open-skill sports depends on the efficient integration of cognitive and motor processes under dual-task (DT) conditions. Reliable measurement of these processes is a prerequisite for assessing the functional profile of young athletes (aged 13–15) and for guiding training.

**Objective:**

This study examined the test-retest reliability (TRR) of center-of-pressure (COP) and fNIRS-derived cortical activation metrics across zero-load, low-load, and high-load conditions, assessed the reliability of N-back performance under the two dual-task conditions, and descriptively compared cortical activation patterns across load conditions in adolescent basketball players.

**Methods:**

Twenty male adolescent basketball players completed a within-subject test-retest protocol involving two visits separated by 7 days. At each visit, participants performed quiet stance alone under the zero-load condition and concurrently with auditory 0-back and 2-back tasks under the low-load and high-load conditions, respectively. Test-retest reliability was quantified using intraclass correlation coefficients (ICCs) for COP indices and fNIRS-derived t-statistics in the bilateral prefrontal cortex (PFC) and motor cortex (MC) across all three load conditions, and for N-back accuracy and reaction time under the two dual-task conditions.

**Results:**

Most indices yielded moderate-to-excellent point estimates of reliability (ICC range: 0.609–0.984), although precision varied, with several 95% confidence intervals spanning multiple reliability categories. COP whole path length, path length per unit area, and the N-back metrics showed good-to-excellent point estimates (ICC ≥ 0.784), and fNIRS-derived t-statistics in the PFC and MC reached ICCs of up to 0.984 under high-load conditions. Within the COP domain, whole path length, circumference area, and path length per unit area constitute three non-redundant indices; mean sway velocity is a duration-normalized expression of whole path length (MSV = WPL/T) and is therefore not counted as separate reliability evidence. Grand-average maps of fNIRS-derived GLM t-statistics appeared qualitatively similar across sessions.

**Conclusion:**

These findings provide preliminary test-retest reliability estimates for selected multimodal neurobehavioral measures in male adolescent basketball players although reliability alone does not establish clinical utility and estimates with wide confidence intervals require confirmation in larger samples. Whether these measures can support monitoring of cognition–action coupling or inform interventions remains to be determined in future validity and responsiveness studies.

## 1 Introduction

In open-skill sports such as basketball, high-level performance depends not only on physical and technical proficiency but also on the ability to flexibly integrate sensorimotor and higher-order cognitive processes under uncertainty – that is, the efficiency of cognition-action coupling under dual-task (DT) constraints (Yaggie and Campbell, 2006; Zech et al., 2010; Lüder et al., 2018; Mancini et al., 2012). Balance control (BC) underpins this coupling. It relies on the central nervous system (CNS) to integrate and reweight multisensory inputs from vision, vestibular, and proprioception in real time (Winter, 1995). Early adolescence (e.g., ages 13–15) is a critical window in which neural networks and executive functions mature rapidly (Paus et al., 2008). Characterizing BC and its neural mechanisms under DT conditions in this population is therefore important both for understanding the functional profile of young athletes and for informing training and injury prevention (Dijkstra et al., 2020; Lüder et al., 2018).

Traditionally, postural control has been quantified using behavioral metrics derived from force plates, particularly center-of-pressure (COP) displacement, velocity, and area. These measures are sensitive to postural sway, discriminate between populations, and can be used for fall-risk evaluation and monitoring (Lafond et al., 2004; Ruhe et al., 2010). Systematic evidence indicates that, under laboratory standardization (e.g., sufficient trial duration and repetitions), many static COP parameters achieve good test-retest reliability (ICC ≥ 0.75). Nonetheless, reliability varies with trial length, number of repetitions, filtering, and analytic choices (Ruhe et al., 2010).

Behavior alone, however, cannot explain how cortical resources are allocated and managed during cognition-action integration. Functional near-infrared spectroscopy (fNIRS) has emerged as a wearable, motion-tolerant, ecologically valid method to monitor cortical hemodynamics in movement settings (Herold et al., 2017; Krougly et al., 2025). By tracking changes in oxygenated (HbO) and deoxygenated hemoglobin (HbR), fNIRS indexes cortical hemodynamic changes associated with neural activity and has been synthesized in consensus guidance for posture and gait research (Herold et al., 2017; Menant et al., 2020). Prior work implicates the prefrontal cortex (PFC) in postural control, sensory reweighting, and DT conflict resolution (Mihara et al., 2008; Monteiro et al., 2024). According to the compensatory recruitment hypothesis, when task demands exceed the processing capacity of specialized neural circuits, the central nervous system mobilizes additional prefrontal resources to sustain behavioral performance (Cabeza, 2002; Reuter-Lorenz and Cappell, 2008). In postural control, this manifests as load-dependent PFC hyperactivation under dual-task conditions, reflecting the reallocation of attentional and executive resources to meet concurrent cognitive and motor demands – a pattern consistently observed across healthy and clinical populations (Boisgontier et al., 2013; St George et al., 2021).

To support athlete monitoring and longitudinal follow-up in youth, the test-retest reliability of these measures is essential. Across tasks and cohorts, fNIRS reliability spans from poor to excellent, shaped by cortical region, task type, processing pipeline, and device/protocol factors such as cap removal. Without cap removal, same-day ICCs in healthy older adults are typically moderate-to-excellent over prefrontal and somatosensory/motor regions; after removal and re-fitting, ICCs decline but generally remain acceptable (Plichta et al., 2007; de Rond et al., 2023). Task paradigm also matters: motor tasks tend to yield higher reliability than passive or cognitive tasks, and block designs outperform event-related designs (Huppert, 2016). Importantly, cortical test-retest reliability has been sparsely studied in youth or athletic populations, and no study has jointly examined the reliability of behavioral, cognitive, and cortical measures within a single dual-task postural paradigm in adolescents.

Compared with adults, adolescent athletes exhibit distinctive characteristics in sensory integration, executive control, and network plasticity, and the the controlled dual-task paradigm approximates selected concurrent cognitive-motor demands relevant to open-skill sports. Although fNIRS test-retest reliability has been systematically characterized in healthy adults, older populations, and clinical cohorts (Plichta et al., 2007; de Rond et al., 2023), the generalizability of these findings to developing athletes remains unclear. Specifically, it is unknown whether the reliability of concurrently measured cortical, cognitive, and behavioral outcomes during dual-task postural control follows age-specific trajectories during the critical neurodevelopmental window of early adolescence – a period during which protracted prefrontal structural remodeling, including synaptic pruning and myelination, may fundamentally alter between-session hemodynamic stability relative to the mature adult brain (Paus et al., 2008). Resolving this uncertainty is a prerequisite for determining whether adult-derived fNIRS reliability benchmarks can be validly extrapolated to longitudinal neurobehavioral monitoring in youth athletes, or whether age-specific normative data must be independently established.

Given that postural reliability depends on trial number, trial duration, and task difficulty (Ruhe et al., 2010; Miner et al., 2022), while fNIRS reliability is sensitive to cap placement, participant-specific factors, and preprocessing choices (Plichta et al., 2007; de Rond et al., 2023), jointly evaluating the test-retest properties of behavior (COP), cognition (N-back performance), and cortical activation (HbO and HbR) within a unified, controlled dual-task paradigm carries both theoretical and applied significance. Such an integrated approach not only addresses the gap in reliability evidence specific to adolescent athletic populations, but also provides a methodological foundation for designing longitudinal training-monitoring protocols that require stable and interpretable neurocognitive metrics. Accordingly, within an integrated neurobehavioral assessment framework, the present study examined the test-retest reliability of COP and fNIRS metrics across zero-load, low-load, and high-load conditions, as well as N-back performance under the two dual-task conditions, in adolescent basketball players. To our knowledge, this is the first study to simultaneously evaluate the test-retest reliability of behavioral, cognitive, and cortical measures within a unified dual-task postural control (DTPC) paradigm in adolescent athletes.

## 2 Methods

### 2.1 Study design and ethics

We conducted a within-subject test-retest study comprising the two laboratory visits were separated by 7 days and were conducted under standardized conditions, including consistent testing time, pre-test activity restrictions, testing environment, and operator procedures. In each visit, participants performed quiet bipedal stance under three load conditions: zero-load, low-load (0-back), and high-load (2-back). The zero-load condition was always completed first, whereas the order of the two dual-task conditions (0-back and 2-back) was counterbalanced within participants. All procedures were approved by the Academic Committee of Capital University of Physical Education and Sports (A2023052) and conformed to the Declaration of Helsinki; written informed consent was obtained from the parents or legal guardians of all participants, and written assent was obtained from each participant prior to participation.

### 2.2 Participants

Twenty male adolescent basketball players were recruited from two Beijing middle schools (age 14.650 ± 0.587 years; education 7.600 ± 0.598 years; height 179.050 ± 7.863 cm; weight 71.875 ± 16.121 kg; BMI 22.222 ± 3.448 kg/m^2^; IPAQ 3446.700 ± 185.190 MET-min/week; MMSE 29.100 ± 0.852; MoCA 28.750 ± 0.716). Inclusion criteria were: 13–15 years; ≥3 years of basketball training; ≥7 h/week training; participation in inter-school competition; and no prior participation in a similar experiment. Prior to testing, demographic, training, and health information was collected; global cognition (MMSE, MoCA) and habitual physical activity (IPAQ-SF) were assessed.

An *a priori* power analysis was performed using G*Power 3.1 (Faul et al., 2007) based on the ICC reliability testing framework proposed by Walter et al. (1998). With the hypotheses H0: ICC ≤ 0.50 versus H1: ICC ≥ 0.80, α = 0.05 (one-tailed), power (1-β) = 0.80, and k = 2 repeated measurements, the minimum required sample size was estimated as *n* = 22. The present study recruited 20 participants, yielding an achieved power of approximately 0.77, slightly below the conventional 0.80 threshold. For reliability studies, however, statistical power is not the primary consideration; the precision of the ICC estimates – reflected in the width of the 95% confidence intervals – is equally important and is governed by the number of participants (Williams et al., 2025; Mondal et al., 2024). This sample size is comparable to or exceeds that of recent fNIRS test-retest reliability studies (e.g., Dong et al., 2024, *n* = 18; Wyser et al., 2022, *n* = 15).

### 2.3 Experimental tasks and apparatus

#### 2.3.1 Balance task and COP acquisition

Balance control was assessed during quiet bipedal stance on a three-dimensional force platform (Fuzhikangda, Beijing), with participants barefoot, feet shoulder-width apart, arms relaxed at the sides, and the body upright. The system sampled at 100 Hz and recorded center-of-pressure (COP) time series. From each trial we derived COP whole path length, circumference area, path length per unit area, and mean sway velocity. These indices were computed in MATLAB (The MathWorks, Inc., Natick, MA) following established posturographic conventions (Prieto et al., 1996; Raymakers et al., 2005; Schubert and Kirchner, 2014): (1) Whole path length (WPL), the total length of the COP trajectory, was computed as the sum of the Euclidean distances between consecutive samples, WPL = $\sum_{i=1}^{N-1}$$\sqrt{\left[{\left(x_{i+1}-x_{i}\right)}^{2}+{\left(y_{i+1}-y_{i}\right)}^{2}\right]}$ larger WPL values indicate greater overall sway and poorer balance control. (2) Circumference area (CFA), the area of the 95% confidence ellipse fitted to the COP data, was computed as CFA = π × a × b, where a and b are the ellipse semi-axes derived from the eigenvalues of the covariance matrix of the COP displacements; larger CFA values indicate a broader sway envelope and poorer balance control. (3) Path length per unit area (PLUA) was computed as the ratio of WPL to CFA (PLUA = WPL/CFA); larger PLUA values indicate a greater COP path length relative to the sway area, which may reflect a long corrective trajectory confined within a small sway area. Because PLUA combines two distinct COP measures rather than rescaling a single measure, its reliability is not mathematically constrained to equal that of either component and was therefore estimated separately. (4) Mean sway velocity (MSV) was computed as MSV = WPL/T, where T = 30 s. Because it is a constant rescaling of WPL, MSV has identical test-retest reliability coefficients and is reported for descriptive comparability only, not as independent reliability evidence. All four indices were computed for each 30-s trial and averaged across the three trials per condition.

#### 2.3.2 Cognitive task (auditory n-back) and dual-tasking

Cognitive load was manipulated with an auditory digit N-back implemented in E-Prime 3.0 (0-back, 2-back). In 0-back, the current digit was compared to “0”; in 2-back, the current digit was compared to the stimulus two positions earlier. Accuracy (ACC) and reaction time (RT) were recorded. All auditory stimuli were delivered as prerecorded audio files spoken by a single speaker and presented at a constant output volume across participants and sessions. Each block began with a 3-s on-screen cue, followed by a 500 ms fixation (“+”), and then a rapid series of digits (0–9), each presented for 500 ms with a 1000 ms inter-stimulus interval (ISI). Each block lasted 30 s and was followed by a 30-s rest period; within 2-back, match/non-match probabilities were equal. For each visit, the digit sequences were randomly generated under the constraint of equal match/non-match probabilities; the test and retest sessions did not use identical sequences; comparable task difficulty was maintained by applying the same sequence-generation constraints across sessions. This randomization was intended to preclude sequence-specific learning and memorization, so that the obtained reliability estimates reflect the stability of the underlying cognitive-motor construct rather than familiarity with a fixed stimulus sequence. Participants completed a 20-trial practice (10 trials per level) before the formal run. During dual-task trials, N-back was performed concurrently with quiet stance; COP and N-back were synchronized, and the task order of 0-back/2-back was counterbalanced within participants.

#### 2.3.3 Trial structure

Per visit, athletes completed three 30-s trials in each load condition (zero-load, low-load, and high-load), with 30-s seated rest between blocks. The mean of the three trials per condition served as the behavioral outcome for that visit. To maintain symmetry in hand loading during responses, participants held an identical second mouse in the non-responding hand.

### 2.4 fNIRS data acquisition

A portable, multi-channel fNIRS system (NirSmartII-3000, Danyang Huichuang Medical Devices, China) measured cortical hemodynamics during all trials. The optode montage followed the international 10–20 system, using 18 sources and 16 detectors (source-detector separation 3 cm) at 780 nm and 830 nm, yielding 42 channels sampled at 10 Hz. Channels were assigned to bilateral prefrontal cortex (PFC; 14 channels) and motor cortex (MC; 28 channels) according to a predefined map; Channel-ROI assignments followed the 10–20 system and are detailed in Supplementary Table 1. To ensure signal quality, gains were adjusted so that gains were adjusted so that channel gain was ≤ 7 prior to recording; channels with a recording gain > 7 were discarded from further analysis prior to recording.

### 2.5 fNIRS preprocessing and outcome derivation

Because fNIRS reflects neural activity indirectly through neurovascular coupling, the term “cortical activation” is used here to refer to task-evoked changes in the fNIRS-derived hemodynamic response (Tachtsidis and Scholkmann, 2016). Preprocessing was conducted in NirSpark (Danyang Huichuang) and MATLAB. Low-quality channels were first identified and excluded based on signal-quality metrics: channels with a signal-to-noise ratio (SNR) < 10 dB during the task period, or with a recording gain exceeding the preset limit (gain > 7), were discarded from further analysis. Motion artifacts were then detected using a moving-window standard deviation criterion (5-s window; threshold of five times the baseline standard deviation) and corrected with spline interpolation (Scholkmann et al., 2010). Data were band-pass filtered at 0.01–0.20 Hz (zero-phase, third-order Butterworth filter) to attenuate slow drift and cardiorespiratory components, and converted from optical density to concentration changes (ΔHbO, ΔHbR, ΔHbT) using the modified Beer-Lambert law. Excluded channels were not imputed; only channels passing quality control in both test and retest sessions were retained, and ROI-level averages were computed over the retained channels of each region (see Supplementary Table 1). Consistent with previous studies using the same multi-channel fNIRS system and vendor software (Chen et al., 2025; An et al., 2025), the preprocessing pipeline was implemented in NirSpark. Baseline correction was then applied by subtracting the mean HbO concentration during the pre-task baseline – the 5-s fixation period immediately preceding each task block – from the HbO time series of the task period: ΔHbO(t) = HbO(t) −$\bar{H\,b\,O}$_*baseline*_, where $\bar{H\,b\,O}$_*baseline*_ = (1/N_*b*_) $\sum_{k=1}^{N_{b}}$ Hbo (t_*k*_) is the mean HbO over the N_b baseline samples (5 s). This yielded the task-related relative change in HbO concentration. To quantify task-evoked activation, a general linear model (GLM) was applied to the baseline-corrected ΔHbO time series of each channel: a boxcar regressor encoding the task on/off periods (30-s task block versus 30-s rest) was convolved with a canonical hemodynamic response function (HRF), and the activation strength of each channel was expressed as a t-statistic, computed as the contrast estimate (task versus baseline) divided by its standard error (t = β/SE(β)). The GLM-derived t-statistic standardizes the contrast estimate by its standard error and therefore incorporates channel-specific uncertainty, although it does not remove between-participant differences in response amplitude. It was therefore used as the outcome for the reliability analysis rather than raw ΔHbO concentration values. For each ROI, the t-statistics of the constituent channels were averaged to obtain the condition-specific ROI-level outcome used in the ICC analyses.

### 2.6 Outcome measures

The behavioral outcomes were COP whole path length, circumference area, path length per unit area, and mean sway velocity (all 100 Hz force-plate derived), plus N-back accuracy (%) and reaction time (ms). The primary neurophysiological outcomes were ROI-level fNIRS-derived t-statistics in bilateral PFC and MC.

### 2.7 Statistical analysis

Analyses were performed in SPSS 29.0 (IBM SPSS, Chicago, IL) and MATLAB. Statistical significance was set at *p* < 0.05 (two-tailed). For test-retest reliability, we computed intraclass correlation coefficients (ICCs) with 95% confidence intervals for each outcome under each cognitive-load condition. Given the test-retest design, in which the same participants were measured on two occasions separated by 7 days, ICCs were calculated using a two-way mixed-effects model with an absolute-agreement definition and average measures (k = 2) – that is, ICC(A,k) following the terminology of McGraw and Wong (1996) and Koo and Li (2016). This model was selected because (1) participants were treated as a random factor and measurement occasions as a fixed factor (two-way mixed-effects model), consistent with the standard implementation of ICC analyses in SPSS and with our original analysis output; (2) absolute agreement rather than consistency was evaluated, because our aim was to determine whether repeated measurements yield comparable absolute values within individuals, which is the question of primary interest for longitudinal monitoring; and (3) average measures (k = 2) rather than single measures were used, because the reliability of interest pertains specifically to the mean of the two assessments obtained in this study. It should be noted that ICC(A,k) is design-specific: the coefficient reported here applies to the mean of two occasions only, and would differ if a different number of occasions were averaged. This average-measures coefficient is therefore reported as the reliability of the two-visit mean in the present protocol, rather than as a general index of longitudinal reliability. Because ICC is invariant to constant rescaling, MSV ( = WPL/T) and WPL yield identical ICCs and 95% confidence intervals; MSV was therefore not treated as independent reliability evidence. PLUA (WPL/CFA), in contrast, combines two distinct COP measures, so its reliability was estimated separately from the observed data. ICC values were interpreted using a four-tier scheme adapted from established interpretation thresholds (Cicchetti, 1994; Koo and Li, 2016; Shrout and Fleiss, 1979): excellent (≥0.90), good (0.75 to <0.90), moderate (0.40 to <0.75), and poor (<0.40). Where a point estimate or a set of estimates spanned more than one tier, the corresponding range is reported explicitly (e.g., good-to-excellent or moderate-to-good). Consistent with the average-measures specification of the ICC, the standard error of measurement (SEM) was computed as SEM = SD_pooled × √[(1 − ICC(A,k))/(k − (k − 1) × ICC(A,k))], where SD_*pooled*_ denotes the pooled standard deviation of the test and retest sessions and k = 2. The smallest detectable change at the 95% confidence level (SDC_95_) was computed as SDC_95_ = 1.96 × √2 × SEM. Pearson correlations were additionally reported to quantify across-session associations.

## 3 Results

### 3.1 Test-retest reliability of balance control indices

In addition to the ICCs, the SEM and SDC_95_ are reported for every index under each load condition (Tables 1, 2, 3), quantifying the absolute measurement error and the smallest detectable change at the 95% confidence level, respectively.

**TABLE 1:** Intraclass correlation coefficients (ICCs) of balance control indices (Mean ± SD).

Index	Load condition	Test (Mean ± SD)	Retest (Mean ± SD)	ICC (95% CI)	SEM	SDC_95_
COP Whole Path Length (mm)	Zero-load	797.17 ± 32.86	795.63 ± 27.08	0.919 (0.810–0.967)	8.24	22.85
	Low-load	844.90 ± 23.89	836.37 ± 19.03	0.820 (0.606–0.924)	8.44	23.38
	High-load	924.05 ± 25.64	920.72 ± 22.00	0.784 (0.538–0.908)	10.07	27.91
COP Circumference Area (mm^2^)	Zero-load	73.92 ± 13.09	75.16 ± 9.53	0.832 (0.629–0.929)	4.34	12.04
	Low-load	102.42 ± 19.13	102.27 ± 17.59	0.609 (0.248–0.823)	9.74	27.01
	High-load	93.07 ± 11.43	96.09 ± 9.83	0.807 (0.581–0.918)	4.29	11.88
COP Path Length per Unit Area (1/mm)	Zero-load	11.06 ± 1.69	10.72 ± 1.21	0.788 (0.545–0.910)	0.61	1.70
	Low-load	8.55 ± 1.75	8.40 ± 1.40	0.857 (0.679–0.940)	0.56	1.55
	High-load	10.05 ± 1.11	9.68 ± 1.03	0.842 (0.649–0.934)	0.40	1.10
COP Mean Sway Velocity (mm/s)	Zero-load	26.57 ± 1.09	26.52 ± 0.90	0.919 (0.810–0.967)	0.27	0.76
	Low-load	28.16 ± 0.80	27.88 ± 0.63	0.820 (0.606–0.924)	0.28	0.78
	High-load	30.80 ± 0.85	30.69 ± 0.73	0.784 (0.538–0.908)	0.34	0.93

ICC(A,k) = intraclass correlation coefficient (two-way mixed-effects model, absolute agreement, average measures, k = 2); CI = confidence interval; SEM = standard error of measurement (SD_pooled × √[(1 − ICC(A,k))/(k − (k − 1) × ICC(A,k))], k = 2); SDC_95_ = smallest detectable change at 95% confidence level (1.96 × √2 × SEM); COP = center of pressure; WPL = whole path length; CFA = circumference area; PLUA = path length per unit area; MSV = mean sway velocity. Because MSV is derived by dividing WPL by the fixed 30-s trial duration, it has identical ICCs and 95% CIs to WPL and is reported descriptively, not as independent reliability evidence. PLUA (WPL/CFA), a ratio of two distinct COP measures, was estimated separately from the observed data. The 95% CIs for ICC(A,k) were calculated using the F-distribution method (Shrout and Fleiss, 1979).

**TABLE 2:** Intraclass correlation coefficients (ICCs) of N-back task accuracy (ACC) and reaction time (RT) (Mean ± SD).

Index	Load condition	Test (Mean ± SD)	Retest (Mean ± SD)	ICC (95% CI)	SEM	SDC_95_
ACC (%)	Low-load	94.40 ± 2.20	94.54 ± 1.74	0.975 (0.939–0.990)	0.31	0.86
	High-load	86.59 ± 3.99	87.55 ± 3.58	0.852 (0.669–0.938)	1.36	3.77
RT (ms)	Low-load	385.08 ± 8.28	382.89 ± 7.94	0.949 (0.878–0.979)	1.79	4.95
	High-load	472.28 ± 9.62	470.58 ± 6.65	0.906 (0.782–0.961)	2.42	6.72

ICC(A,k) = intraclass correlation coefficient (two-way mixed-effects model, absolute agreement, average measures, k = 2); CI = confidence interval; SEM = standard error of measurement (SD_pooled × √[(1 − ICC(A,k))/(k − (k − 1) × ICC(A,k))], k = 2); SDC_95_ = smallest detectable change at 95% confidence level (1.96 × √2 × SEM); ACC = accuracy; RT = reaction time. The 95% CIs for ICC(A,k) were calculated using the F-distribution method (Shrout and Fleiss, 1979).

**TABLE 3:** Intraclass correlation coefficients (ICCs) of fNIRS-derived t-statistics in the prefrontal cortex (PFC) and motor cortex (MC) (Mean ± SD).

Cortical region	Load condition	Test (Mean ± SD)	Retest (Mean ± SD)	ICC (95% CI)	SEM	SDC_95_
Right PFC	Zero-load	1.21 ± 1.49	0.89 ± 1.47	0.977 (0.944–0.991)	0.22	0.62
	Low-load	1.99 ± 1.54	1.79 ± 1.40	0.936 (0.848–0.974)	0.36	1.00
	High-load	0.44 ± 1.32	0.36 ± 1.61	0.984 (0.961–0.994)	0.18	0.51
Left PFC	Zero-load	1.17 ± 1.39	1.14 ± 1.50	0.682 (0.361–0.860)	0.71	1.97
	Low-load	2.06 ± 1.44	1.78 ± 1.30	0.958 (0.899–0.983)	0.28	0.76
	High-load	1.34 ± 1.31	1.12 ± 1.28	0.982 (0.956–0.993)	0.17	0.48
Right MC	Zero-load	1.37 ± 1.56	1.21 ± 1.37	0.850 (0.665–0.937)	0.53	1.47
	Low-load	1.45 ± 1.55	1.57 ± 1.32	0.899 (0.767–0.958)	0.44	1.21
	High-load	0.81 ± 1.48	1.15 ± 1.24	0.836 (0.511–0.947)	0.51	1.42
Left MC	Zero-load	1.17 ± 1.76	1.42 ± 1.79	0.877 (0.720–0.949)	0.59	1.63
	Low-load	1.33 ± 1.70	1.35 ± 1.40	0.937 (0.851–0.974)	0.38	1.05
	High-load	1.58 ± 1.38	1.21 ± 1.42	0.948 (0.876–0.979)	0.31	0.86

ICC(A,k) = intraclass correlation coefficient (two-way mixed-effects model, absolute agreement, average measures, k = 2); CI = confidence interval; SEM = standard error of measurement (SD_pooled × √[(1 − ICC(A,k))/(k − (k − 1) × ICC(A,k))], k = 2); SDC_95_ = smallest detectable change at 95% confidence level (1.96 × √2 × SEM); PFC = prefrontal cortex; MC = motor cortex. fNIRS outcomes are expressed as t-statistics derived from the GLM contrast between task and baseline. The 95% CIs for ICC(A,k) were calculated using the F-distribution method (Shrout and Fleiss, 1979).

Table 1 summarizes the test-retest reliability of center-of-pressure (COP) parameters across three cognitive-load conditions in adolescent basketball players. WPL showed good-to-excellent reliability (ICC = 0.784–0.919). MSV showed identical estimates because it is a constant rescaling of WPL (MSV = WPL/T) and was therefore not interpreted as independent reliability evidence. PLUA also showed good reliability (ICC = 0.788–0.857); its point estimate was the highest among the three COP indices under both dual-task loads but the lowest under zero-load conditions, although these differences were not formally tested and the confidence intervals overlapped. As a ratio of WPL to CFA, PLUA provides non-redundant reliability information and was retained as a distinct COP measure. COP circumference area produced the least precise estimates: its ICC point estimates ranged from 0.609 to 0.832, and the 95% CI for the low-load condition (0.248–0.823) spanned the poor-to-good range, indicating substantial uncertainty. More generally, several confidence intervals crossed the 0.75 threshold (e.g., WPL under high-load conditions: 95% CI 0.538–0.908), so the corresponding point estimates should be interpreted as preliminary rather than definitively graded. Pearson correlation analyses showed significant positive associations between test and retest values for all COP indices under zero-load, low-load, and high-load conditions (all p < 0.05; Supplementary Figure 1A).

### 3.2 Test-retest reliability of cognitive performance indices

Table 2 presents the test-retest reliability of N-back accuracy (ACC) and reaction time (RT) under the low-load (0-back) and high-load (2-back) conditions. Both measures yielded good-to-excellent point estimates across the two dual-task conditions (ICC = 0.852–0.975); however, the 95% CI for 2-back ACC (0.669–0.938) spanned the moderate-to-excellent range, indicating uncertainty in the reliability classification. Pearson correlations showed strong positive associations between test and retest scores for both ACC and RT under the two conditions (all *p* < 0.001; Supplementary Figure 1B).

### 3.3 Test-retest reliability of fNIRS outcomes

Intraclass correlation coefficients for fNIRS-derived t-statistics in bilateral prefrontal cortex (PFC) and motor cortex (MC) are summarized in Table 3. Under zero-load conditions, right PFC t-statistics showed excellent point estimates (ICC = 0.977), whereas left PFC yielded only moderate point estimates (ICC = 0.682) whose 95% CI lower bound fell below 0.40 (95% CI 0.361–0.860), warranting conservative interpretation. Right and left MC demonstrated good point estimates (ICC = 0.850 and 0.877, respectively).

Under low-load conditions, both left and right PFC showed excellent point estimates (ICC = 0.936–0.958), and bilateral MC showed good-to-excellent point estimates (ICC = 0.899–0.937). Under high-load conditions, PFC exhibited excellent point estimates bilaterally (ICC = 0.982–0.984), with narrow confidence intervals (95% CI 0.956–0.994), whereas MC yielded good-to-excellent point estimates (ICC = 0.836–0.948).

Pearson correlation analyses (Supplementary Figure 1C) showed significant positive associations between test and retest values in most regions, with the strongest correlations observed in bilateral PFC under high-load conditions (r = 0.976–0.984, all *p* < 0.001). Moreover, grand-average cortical activation maps across sessions (Figure 1) appeared qualitatively similar across sessions; however, no quantitative map-level reproducibility analysis was performed across all load conditions, with bilateral PFC exhibiting the highest intersession reproducibility under high-load conditions (ICC = 0.982–0.984), consistent with the quantitative ICC pattern reported in Table 3.

**FIGURE 1:**
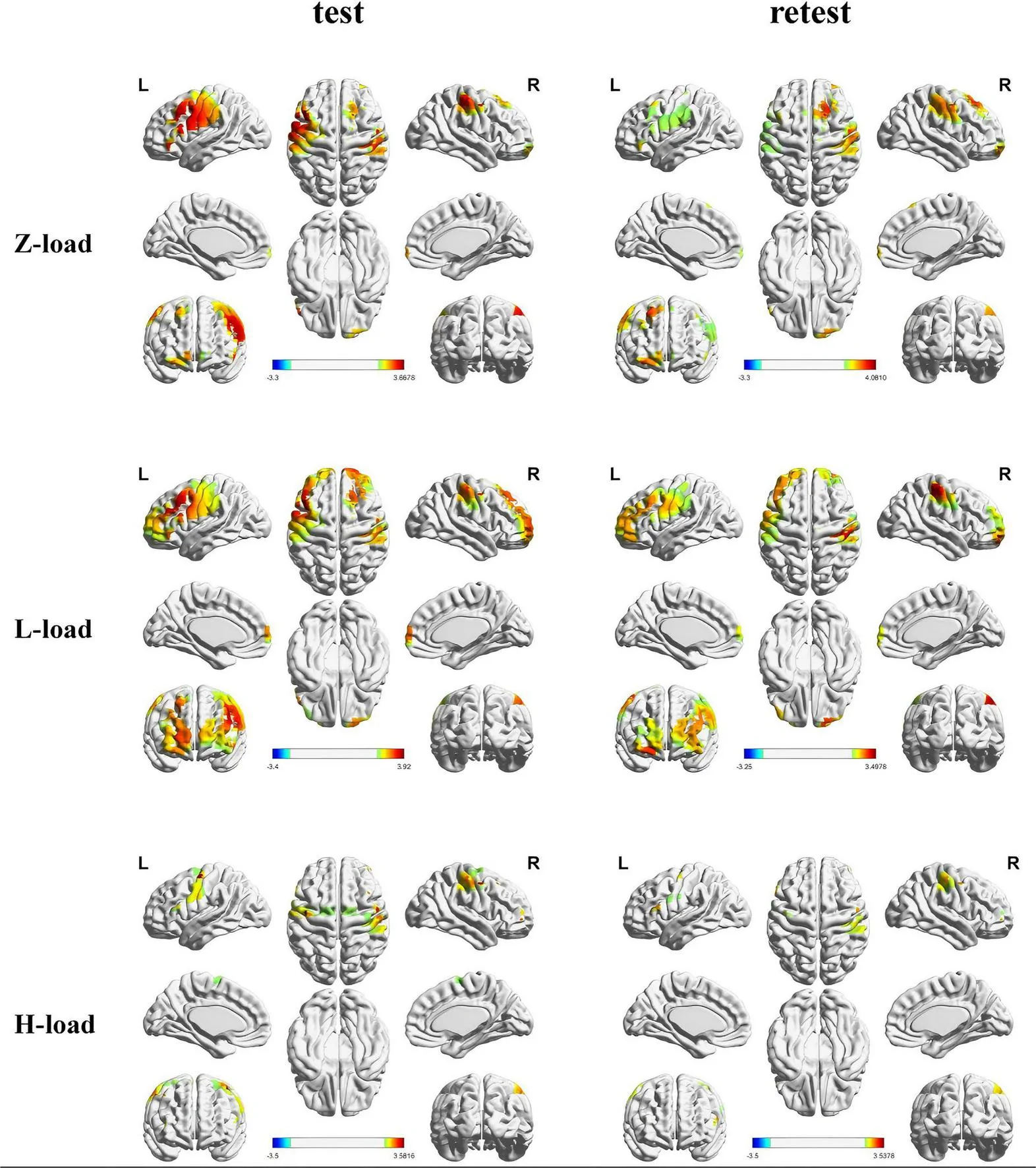
Grand-average brain activation maps across cognitive-load conditions during test and retest in adolescent basketball players (ages 13–15). Z-load = zero-load; L-load = low-load; H-load = high-load; Test = first session; Retest = second session.

## 4 Discussion

This study evaluated the test-retest reliability of postural (COP), cognitive (N-back), and cortical activation (fNIRS-derived t-statistics) measures in adolescent basketball players during a DTPC paradigm. Most indices showed moderate-to-excellent ICC point estimates (0.609–0.984), although several confidence intervals spanned multiple reliability categories, warranting cautious interpretation. Overall, these findings provide preliminary evidence that selected ROI-level fNIRS-derived t-statistics show moderate-to-excellent ICC point estimates in this controlled quiet-stance dual-task paradigm, providing methodological support for future studies that aim to use these metrics to assess balance function or track training-induced neural adaptations (Menant et al., 2020; Herold et al., 2017).

### 4.1 Reliability of behavioral indices and insights into postural control mechanisms

COP parameters across the three load conditions and N-back performance under the two dual-task conditions generally exhibited moderate-to-excellent point estimates of reliability. However, estimates with wide confidence intervals, particularly CFA under low-load conditions, should be considered preliminary pending confirmation in larger samples. This pattern is broadly consistent with systematic evidence on static postural measures (Ruhe et al., 2010). Within the COP domain, three non-redundant indices were examined: WPL reflects the overall COP trajectory, CFA represents the spatial extent of sway, and PLUA describes path length relative to sway area. WPL showed good-to-excellent reliability and PLUA showed good reliability, whereas CFA showed greater variation in reliability estimates across load conditions. Because MSV was calculated as WPL divided by the fixed 30-s trial duration, it yielded identical reliability estimates and did not constitute independent reliability evidence. Although PLUA showed numerically higher ICC point estimates under dual-task conditions, the overlapping confidence intervals and absence of formal comparisons preclude conclusions of improved reliability. Averaging three 30-s trials was intended to reduce within-session variability and improve estimation precision; a comparable protocol has been used in an fNIRS study of postural control (Liu et al., 2024). SEM and SDC_95_ were also reported to characterize measurement error on the two-occasion average-measures scale (Liljequist et al., 2019; Niu et al., 2013).

### 4.2 Reliability of cortical activation measures

A key finding on the neuroimaging front was the moderate-to-excellent point estimates of test-retest reliability for fNIRS-derived t-statistics in the prefrontal cortex (PFC) and motor-related cortices; as with the behavioral indices, several regional estimates were associated with wide confidence intervals (e.g., left PFC under zero-load conditions) and should therefore be interpreted with caution. This is consistent with recent fNIRS-TRR research demonstrating that moderate-to-excellent reliability in the PFC and somatosensory-motor regions can be achieved without cap removal or with strictly controlled refitting across short-term repeated measures; however, reliability may decline following cap removal and refitting, with motor-related areas (e.g., SMA/PMC) being particularly sensitive (Ranchet et al., 2023; de Rond et al., 2023). Mechanistically, the higher point estimates for PFC signals under high-load conditions may be consistent with the compensatory recruitment, or task-locking, account: as task difficulty and conflict-control demands increase, PFC involvement in attentional maintenance and interference suppression may become more sustained and patterned, which could in turn yield a more stable spatiotemporal trajectory across repeated measures (Herold et al., 2017; Mihara et al., 2008). By contrast, motor cortex signals may be more susceptible to slight postural movements and subtle changes in cap position, explaining their greater decline in between-day reliability (de Rond et al., 2023). Therefore, adequate reliability is necessary for distinguishing change from measurement error, but reliability alone does not establish that an observed change is a true adaptation in intervention studies where neural efficiency is hypothesized.

An alternative, non-mutually exclusive interpretation should also be considered: conceptually, the ICC reflects between-participant variance relative to total variance, although the exact variance components depend on the selected ICC model. Consequently, factors that increase between-participant variability relative to measurement error can increase ICC estimates (Bartko, 1966). Under high-load conditions, the contribution of signal strength to the high-load ICCs cannot be determined because raw HbO amplitudes and task-evoked SNR were not reported, magnifying inter-individual differentiation in hemodynamic response magnitude (Wyser et al., 2022). It is therefore conceivable that elevated PFC ICCs partially reflect enhanced between-subject discriminability driven by signal strength rather than genuinely improved within-subject stability. However, three considerations do not exclude an SNR-based explanation. First, the present analyses used GLM-derived t-statistics, which partly account for uncertainty by scaling the contrast estimate by its standard error and thereby limit, although do not eliminate, absolute amplitude scaling as a source of between-subject variance; the ICC estimates therefore describe the consistency of the standardized contrast rather than raw signal magnitude alone. Second, a generalized SNR effect would predict uniformly elevated ICCs across cortical regions under high-load conditions, yet the gain was not uniform: ICCs increased substantially in the left PFC and left MC, whereas the right PFC and right MC showed no comparable improvement (Table 3), which is inconsistent with a global SNR account. Third, the task-locking model, grounded in PFC functional specialization for attentional maintenance and interference suppression (Mihara et al., 2008; Herold et al., 2017), predicts the anatomically constrained reliability enhancement we observed, whereas a generic SNR mechanism makes no region-specific predictions. Future studies employing within-subject coefficient of variation, multivariate pattern reproducibility, or task-evoked SNR from GLM residuals (Huppert, 2016) are warranted to quantitatively dissociate signal stability from signal strength in fNIRS test-retest reliability.

### 4.3 Reliability patterns across cognitive-load conditions

We observed a region-dependent pattern of reliability rather than a uniform load-dependent gradient. In the left PFC, the reliability of fNIRS-derived t-statistics was lower under zero-load than dual-task conditions (0.682 vs. 0.958 and 0.982), and the same ordering was evident in the SEM and SDC_95_ values for this region; the left MC showed a comparable monotonic increase (0.877, 0.937, and 0.948). The right-hemisphere regions did not follow this ordering: the weakest estimate for the right PFC occurred under low-load conditions (0.936; zero-load = 0.977, high-load = 0.984), and for the right MC under high-load conditions (0.836; zero-load = 0.850, low-load = 0.899). Because ICCs were not formally compared across load conditions, these regional differences are descriptive and do not in themselves constitute evidence of a load effect. The term “task-locking-stability increase” is used as a working description of the left-hemisphere pattern: absent external cognitive constraints, PFC hemodynamics are susceptible to intrinsic fluctuations (e.g., mind-wandering, default mode activity) that inflate between-session measurement variance, whereas increasing task demands may progressively constrain these fluctuations and yield more temporally consistent activation patterns across repeated measurements (Plichta et al., 2007; Herold et al., 2017).

A conceptually parallel phenomenon has been observed in clinical populations: during postural control, individuals with chronic low back pain show greater dorsolateral prefrontal activation than controls, with activation increasing as stance difficulty rises (Li et al., 2023), whereas individuals with chronic ankle instability show increased activation in sensorimotor regions (M1, PMC/SMA, and S1) that is associated with poorer lateral balance control (Liu et al., 2024). It must be emphasized, however, that the functional significance differs fundamentally: in clinical populations, this over-recruitment of cortical resources reflects compensation for impaired peripheral or subcortical systems (a functional deficit), whereas in healthy adolescent athletes, prefrontal engagement under dual-task load may represent a hypothesis for future testing during an active phase of motor skill acquisition and prefrontal structural remodeling (Paus, 2005). Notwithstanding this distinction, a shared operating principle may link both phenomena: when task demands encroach upon processing capacity limits, prefrontal engagement becomes more temporally constrained and spatially patterned, yielding greater activation stability across repeated measurements. This “load-locking” mechanism – whether driven by pathological necessity or adaptive skill refinement – may represent a domain-general property of prefrontal function under high cognitive-motor demand. The present findings provide indirect, reliability-based evidence and do not constitute formal validation of this framework. Critical future steps include formal comparison of dependent ICCs across load conditions, direct decomposition of between- and within-participant variance, together with task-evoked SNR analyses, to distinguish these competing explanations.

### 4.4 Methodological implications and future research directions

By establishing test-retest reliability for COP, N-back, and fNIRS-derived t-statistics in adolescent basketball players, the present study provides a methodological prerequisite for the future application of these measures. Reliability evidence alone, however, does not establish what a measure can be used for; injury-risk screening, training prescription, and intervention evaluation require validity, predictive, and responsiveness evidence that lie beyond the scope of this study. Whether these metrics can ultimately support such applications therefore remains an open question for future research. Future work should proceed along three primary paths: first, procedural and statistical optimization – preregister the protocol and report trial-order sensitivity analyses (for example, excluding the first trial) to assess whether reliability estimates may be influenced by residual practice effects, and adhere to standard reporting of ICC model, SEM, SDC, and Bland-Altman plots (de Rond et al., 2023; Liljequist et al., 2019; Niu et al., 2013); second, expansion of metrics – incorporate TRR assessment for functional connectivity (FC) and network topology indices, given recent evidence on the repeatability of fronto-parietal networks during walking tasks, advancing from “local activation” to “network coupling” (Dong et al., 2024); third, short-channel regression – following consensus recommendations, incorporate short-separation channels as a standard preprocessing step to enhance physiological specificity (Menant et al., 2020; Yücel et al., 2021).

## 5 Limitations

Several limitations of the present study should be acknowledged. Although this work provides test-retest reliability estimates for concurrently recorded postural (COP), cognitive (N-back), and cortical (fNIRS-derived t-statistics) metrics under systematically varied dual-task demands in adolescent athletes, the following caveats warrant consideration when interpreting and extending these findings.

First, the sample was restricted to male adolescent basketball players, limiting the generalizability of the reported reliability benchmarks to female athletes and practitioners of other sport disciplines. Basketball imposes unique postural demands – jump-landing stabilization, rapid directional changes, and upper-limb interference during balance – that may recruit distinct cortical networks and dual-task strategies not directly transferable to sports with contrasting postural-motor profiles, such as gymnastics or soccer. Whether the moderate-to-excellent ICCs observed here generalize across sex and sport type remains an open empirical question; replication in diverse athletic populations is needed to establish the boundary conditions of these benchmarks.

Second, the 7-day test-retest interval, though consistent with common practice in fNIRS reliability research (Plichta et al., 2007; Blasi et al., 2014), may not have fully eliminated learning effects specific to the auditory N-back task. Repeated N-back practice may produce task-specific learning effects (Jaeggi et al., 2008), which could partially conflate practice-driven stability with true test-retest reliability. Notably, the randomization of digit sequences across sessions rules out sequence-specific memorization as a source of inflated reliability; nonetheless, generic strategy consolidation may still occur and could partially influence the test-retest estimates. The counterbalanced task-order design and pre-experimental practice trials may have mitigated this concern. However, high ICCs do not exclude practice effects. Nonetheless, future studies should consider extended intersession intervals (≥14 days) or alternative executive-control tasks less prone to procedural learning.

Third, the fNIRS preprocessing pipeline did not incorporate short-channel regression to attenuate systemic hemodynamic contributions originating from superficial scalp layers (Huppert, 2016; Menant et al., 2020). Without this correction, a fraction of the measured ΔHbO may reflect extracerebral sources – scalp blood flow, blood pressure oscillations, and respiration-related fluctuations – rather than task-evoked cortical activity (Tachtsidis and Scholkmann, 2016), potentially inflating or attenuating ICCs in a region- and condition-dependent manner. The extent to which the present reliability estimates reflect cortical as opposed to systemic contributions therefore cannot be fully established. Following consensus recommendations (Menant et al., 2020; Yücel et al., 2021), future work should incorporate short-separation channels as a standard preprocessing step to enhance physiological specificity.

Fourth, the achieved sample (*n* = 20) fell slightly below the *a priori* estimate of *n* = 22 derived from the ICC reliability testing framework, yielding an achieved power of approximately 0.77. Sample size directly governs the precision of ICC estimates, because the width of the 95% confidence interval (CI) narrows as the number of participants increases (Williams et al., 2025; Mondal et al., 2024). This effect was most evident for the COP circumference area under low-load conditions (ICC = 0.609), whose 95% CI spanned 0.248–0.823 (width ≈ 0.58), whereas indices with higher ICCs or lower measurement variability yielded substantially narrower intervals. It should nevertheless be noted that the sample size required for a reliability study depends on the anticipated ICC magnitude and the desired CI width rather than on a fixed threshold (Mondal et al., 2024; Williams et al., 2025); assuming an expected ICC ≥ 0.75, the although the sample size was comparable to those of some recent fNIRS reliability studies, it was below the prespecified target of 22, and several outcomes were estimated with wide confidence intervals (e.g., Dong et al., 2024, *n* = 18; Wyser et al., 2022, *n* = 15). Accordingly, two tiers of conclusions should be distinguished: (i) findings supported by relatively precise estimates – most COP measures, N-back outcomes, and regional and condition-specific fNIRS-derived t-statistics showed moderate-to-excellent reliability with reasonably narrow confidence intervals; and (ii) findings warranting cautious interpretation – notably CFA under low-load conditions and left PFC t-statistics under zero-load conditions, both of which had wide confidence intervals with lower bounds below 0.40. Replication with larger samples remains warranted to narrow the confidence intervals of these less stable indices and to confirm the region- and load-dependent reliability patterns.

Fifth, the present paradigm was limited to static bipedal stance, which captures only one dimension of postural control. Dynamic balance tasks – such as single-leg standing, perturbation responses, and sport-specific maneuvers – engage distinct sensorimotor processes and may yield different test-retest reliability profiles (Menant et al., 2020). Extending the multimodal framework to ecologically valid, movement-based paradigms is an important direction for evaluating its applicability to athlete monitoring.

## 6 Conclusion

These findings provide preliminary evidence for the test-retest reliability of several multimodal neurobehavioral measures in male adolescent basketball players. The variation in precision across outcomes underscores the importance of considering both ICC point estimates and their confidence intervals; measures supported by relatively precise estimates may be better suited to longitudinal research, whereas imprecise estimates require confirmation in larger samples. Overall, this framework provides a methodological basis for investigating cognition-action coupling and training-related adaptations, but further validation in larger, more diverse samples and dynamic, sport-specific contexts is warranted.

## Data Availability

The raw data supporting the conclusions of this article will be made available by the authors, without undue reservation.
