# Supplementary material for: The test-retest reliability of cortical activation and behavioral metrics in adolescent basketball players during dual-task postural control: an fNIRS-based assessment
Source: Front Psychol. 2026 Oct 9;17:1948562. doi: 10.3389/fpsyg.2026.1948562 (PMC13690641; doi:10.3389/fpsyg.2026.1948562)
Supplement: Supplementary file 1 [file Supplementary_file_1.pdf]

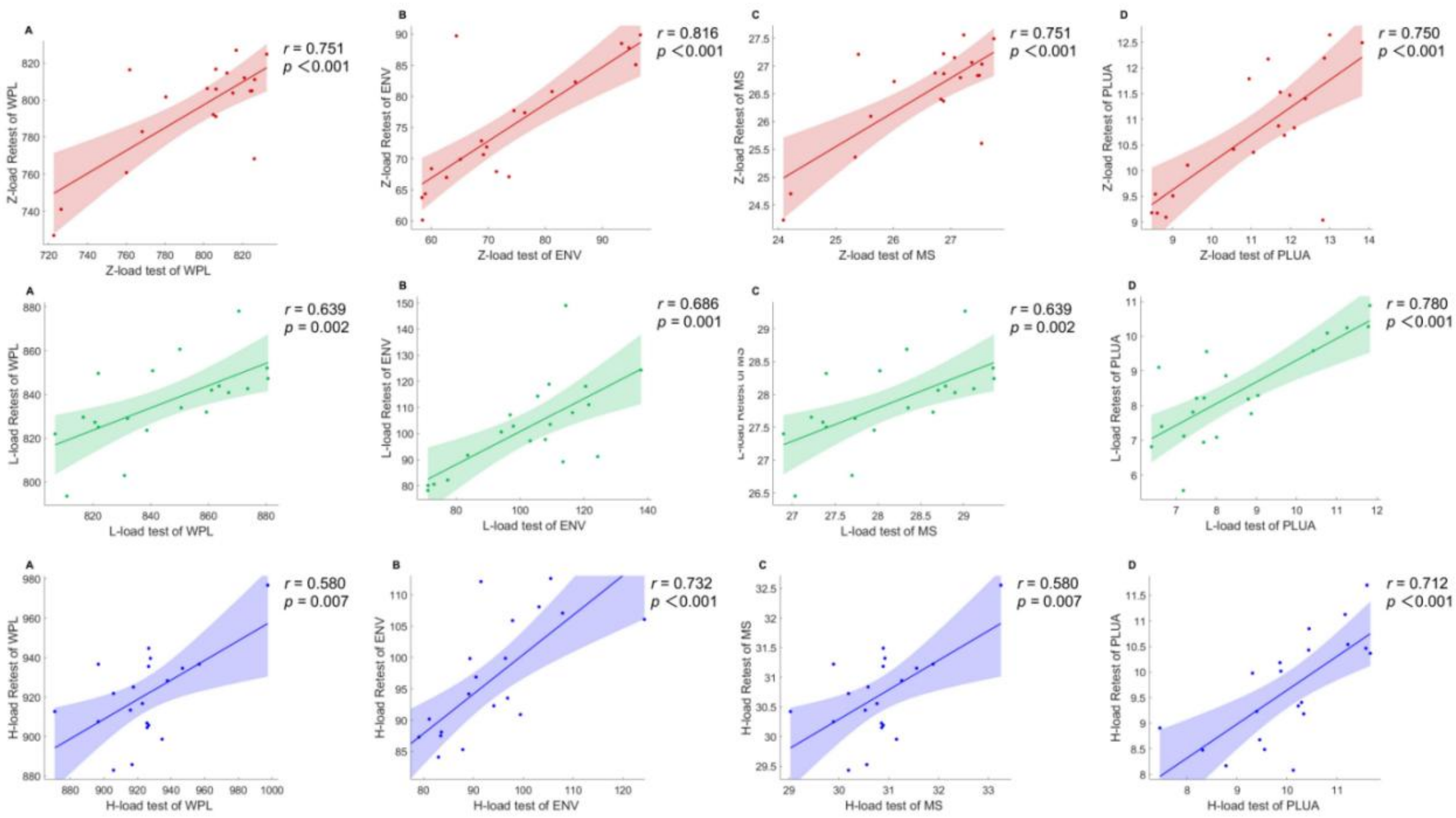

A
Z-load Retest of WPL
Z-load test of WPL
r = 0.751
p < 0.001
B
Z-load Retest of ENV
Z-load test of ENV
r = 0.816
p < 0.001
C
Z-load Retest of MS
Z-load test of MS
r = 0.751
p < 0.001
D
Z-load Retest of PLUA
Z-load test of PLUA
r = 0.750
p < 0.001
A
L-load Retest of WPL
L-load test of WPL
r = 0.639
p = 0.002
B
L-load Retest of ENV
L-load test of ENV
r = 0.686
p = 0.001
C
L-load test of MS
r = 0.639
p = 0.002
D
L-load Retest of PLUA
L-load test of PLUA
r = 0.780
p < 0.001
A
H-load Retest of WPL
H-load test of WPL
r = 0.580
p = 0.007
B
H-load Retest of ENV
H-load test of ENV
r = 0.732
p < 0.001
C
H-load Retest of MS
H-load test of MS
r = 0.580
p = 0.007
D
H-load Retest of PLUA
H-load test of PLUA
r = 0.712
p < 0.001

A

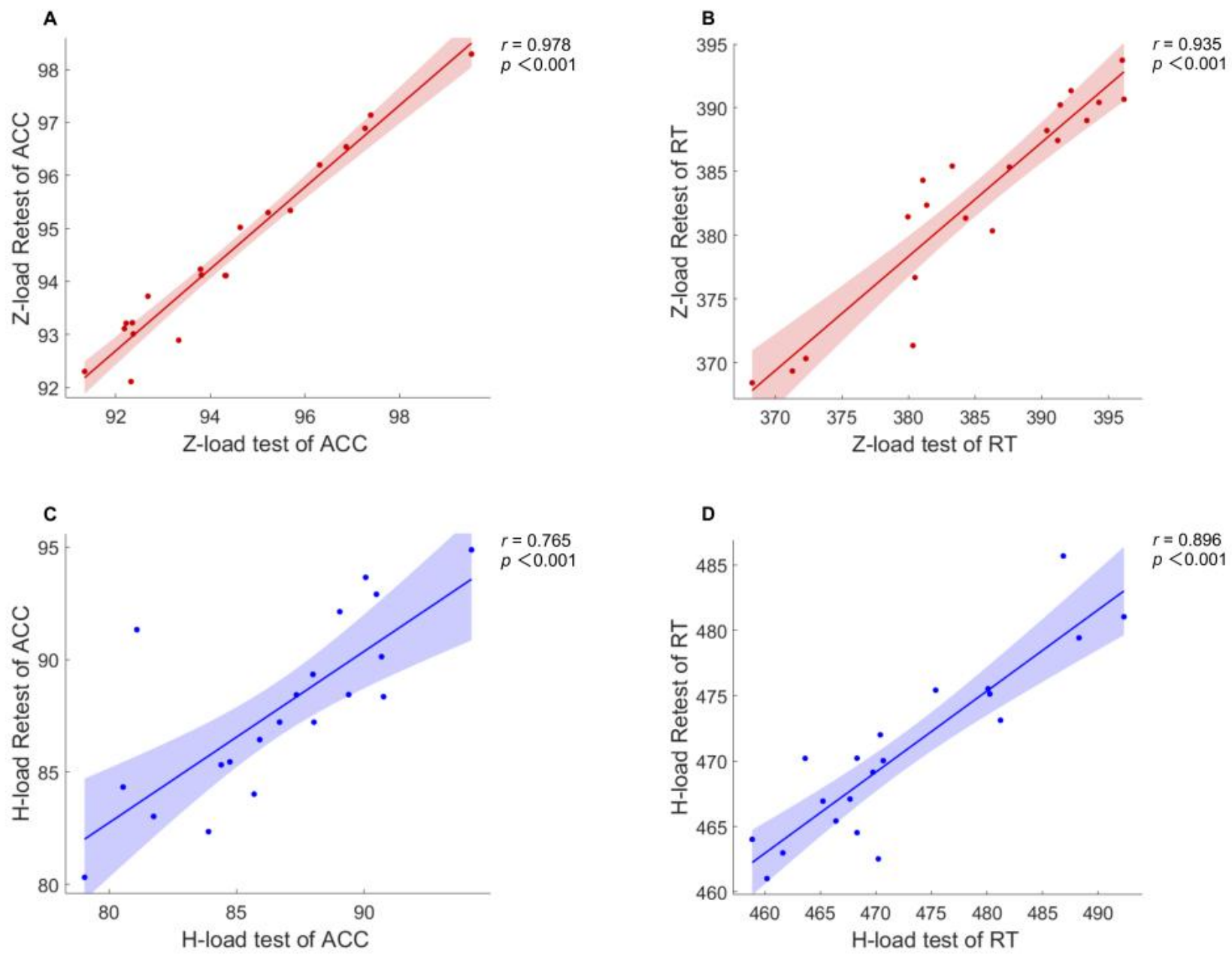

A
Z-load Retest of ACC
Z-load test of ACC
r = 0.978
p < 0.001
B
Z-load Retest of RT
Z-load test of RT
r = 0.935
p < 0.001
C
H-load Retest of ACC
H-load test of ACC
r = 0.765
p < 0.001
D
H-load Retest of RT
H-load test of RT
r = 0.896
p < 0.001

B

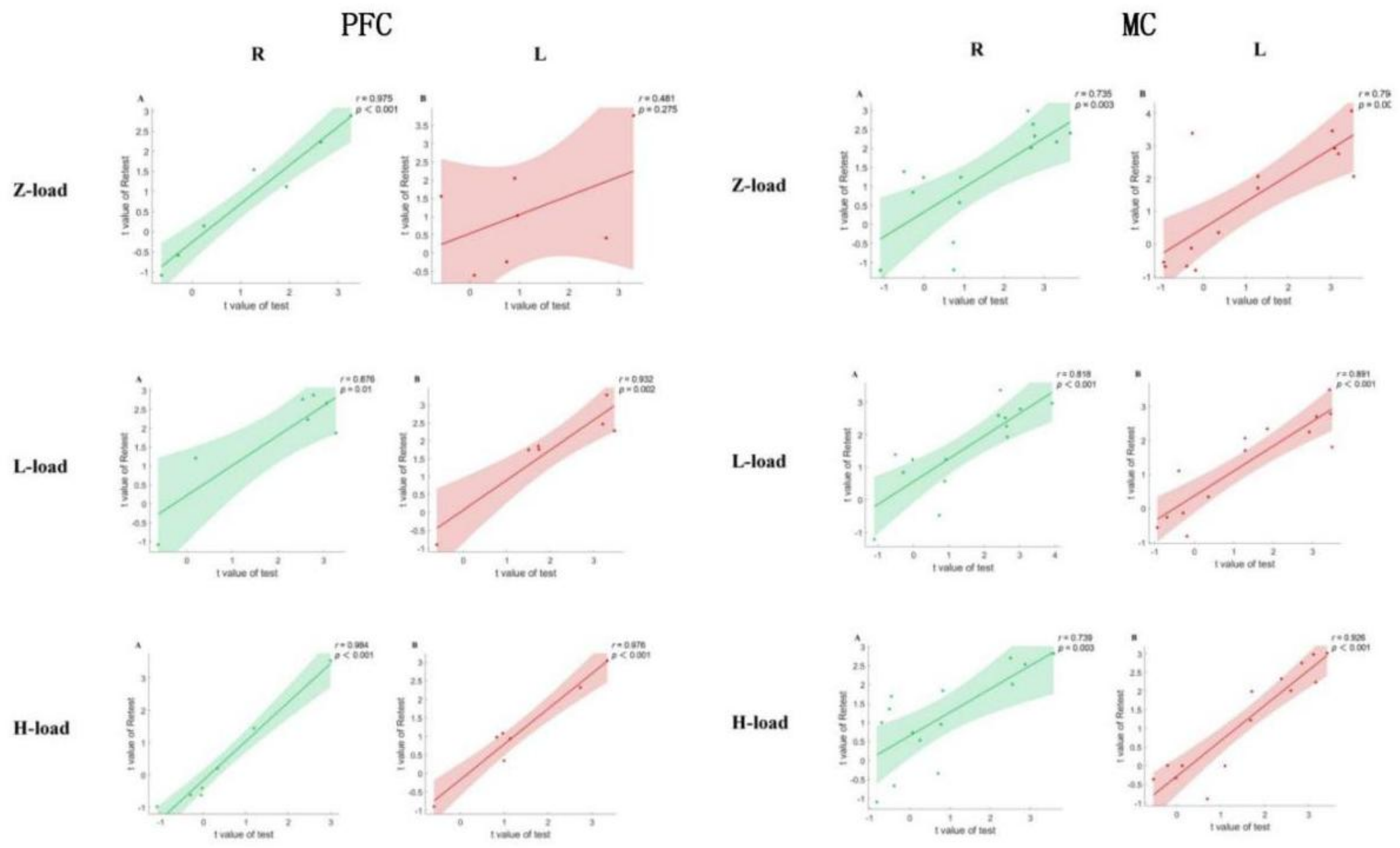

C

Supplementary Figure S1. Pearson correlation analyses of test and retest values, presented as a single multi-panel figure. Panel A: balance control indices (COP whole path length, circumference area, path length per unit area, and mean sway velocity); Panel B: N-back task accuracy (ACC) and reaction time (RT); Panel C: fNIRS-derived t-statistics in the prefrontal cortex (PFC) and motor cortex (MC).
