# Supplementary material for: The test-retest reliability of cortical activation and behavioral metrics in adolescent basketball players during dual-task postural control: an fNIRS-based assessment
Source: Front Psychol. 2026 Oct 9;17:1948562. doi: 10.3389/fpsyg.2026.1948562 (PMC13690641; doi:10.3389/fpsyg.2026.1948562)
Supplement: Supplementary file 2 [file Data_Sheet_1.docx]

Supplementary Table S1

Channel positions and cortical region assignments for the 42-channel fNIRS montage

Montage configuration: 18 sources × 16 detectors, source–detector separation = 3 cm, 42 channels. Channels were assigned to four ROIs based on their corresponding Brodmann areas (BA) and MNI coordinates determined via the Talairach daemon registration (NirSpark built-in probe registration).

ROI legend: R-PFC = right prefrontal cortex; L-PFC = left prefrontal cortex; R-MC = right motor cortex; L-MC = left motor cortex.

| **Channel** | **S-D Pair** | **ROI** | **Primary Cortical Region (BA)** | **X (mm)** | **Y (mm)** | **Z (mm)** |
| --- | --- | --- | --- | --- | --- | --- |
| CH1 | S1-D1 | R-PFC | BA 47 (Inferior prefrontal gyrus) | -56.044 | 31.877 | -8.554 |
| CH2 | S1-D3 | R-PFC | BA 45/46 (Pars triangularis / DLPFC) | -54.542 | 38.051 | 13.665 |
| CH3 | S2-D2 | R-MC | BA 6 (Pre-motor & SMA) | -67.121 | -5.288 | 31.32 |
| CH4 | S2-D4 | R-MC | BA 9/8 (DLPFC / Frontal eye fields) | -53.898 | 20.769 | 39.554 |
| CH5 | S2-D5 | R-MC | BA 6 (Pre-motor & SMA) | -58.877 | -5.449 | 48.737 |
| CH6 | S3-D2 | R-MC | BA 40 (Supramarginal gyrus) | -68.765 | -31.573 | 37.993 |
| CH7 | S3-D5 | R-MC | BA 2/1/40 (S1 / Supramarginal gyrus) | -57.577 | -28.063 | 54.707 |
| CH8 | S4-D1 | R-PFC | BA 11/47 (Orbitofrontal / Inferior PFC) | -49.995 | 45.658 | -17.639 |
| CH9 | S4-D3 | R-PFC | BA 10 (Frontopolar area) | -48.077 | 54.789 | -0.361 |
| CH10 | S4-D6 | R-PFC | BA 11/10 (Orbitofrontal / Frontopolar) | -35.545 | 64.34 | -13.343 |
| CH11 | S5-D4 | R-MC | BA 8 (Frontal eye fields) | -43.982 | 21.893 | 51.564 |
| CH12 | S5-D5 | R-MC | BA 6 (Pre-motor & SMA) | -46.453 | -2.345 | 59.066 |
| CH13 | S5-D7 | R-MC | BA 8/6 (Frontal eye fields / Pre-motor) | -33.77 | 21.177 | 60.132 |
| CH14 | S5-D8 | R-MC | BA 6 (Pre-motor & SMA) | -35.386 | -3.077 | 65.833 |
| CH15 | S6-D5 | R-MC | BA 3/1/2 (Primary S1) | -48.717 | -27.556 | 66.048 |
| CH16 | S6-D8 | R-MC | BA 3/4/6 (S1 / M1 / Pre-motor) | -36.322 | -26.319 | 71.502 |
| CH17 | S7-D3 | R-PFC | BA 10 (Frontopolar area) | -37.554 | 58.619 | 20.856 |
| CH18 | S7-D6 | R-PFC | BA 10 (Frontopolar area) | -25.503 | 70.715 | 4.906 |
| CH19 | S8-D7 | R-MC | BA 6 (Pre-motor & SMA) | -20.019 | 21.001 | 67.148 |
| CH20 | S8-D8 | R-MC | BA 6 (Pre-motor & SMA) | -23.346 | -3.289 | 75.594 |
| CH21 | S9-D8 | R-MC | BA 6/4 (Pre-motor / M1) | -24.42 | -24.684 | 76.414 |
| CH22 | S10-D10 | L-MC | BA 6 (Pre-motor & SMA) | 22.866 | 23.149 | 65.084 |
| CH23 | S10-D11 | L-MC | BA 6 (Pre-motor & SMA) | 23.386 | -2.004 | 75.113 |

| **Channel** | **S-D Pair** | **ROI** | **Primary Cortical Region (BA)** | **X (mm)** | **Y (mm)** | **Z (mm)** |
| --- | --- | --- | --- | --- | --- | --- |
| CH24 | S11-D11 | L-MC | BA 4/6/3 (M1 / Pre-motor / S1) | 23.215 | -25.161 | 76.618 |
| CH25 | S12-D9 | L-PFC | BA 10 (Frontopolar area) | 29.335 | 67.64 | 8.043 |
| CH26 | S12-D12 | L-PFC | BA 10/9 (Frontopolar / DLPFC) | 38.566 | 55.986 | 22.961 |
| CH27 | S13-D10 | L-MC | BA 8/6 (Frontal eye fields / Pre-motor) | 32.821 | 22.305 | 59.685 |
| CH28 | S13-D11 | L-MC | BA 6 (Pre-motor & SMA) | 33.636 | -4.17 | 66.606 |
| CH29 | S13-D13 | L-MC | BA 8 (Frontal eye fields) | 42.679 | 22.374 | 52.733 |
| CH30 | S13-D14 | L-MC | BA 6 (Pre-motor & SMA) | 43.756 | -1.43 | 61.243 |
| CH31 | S14-D11 | L-MC | BA 4/6/3 (M1 / Pre-motor / S1) | 34.317 | -25.306 | 72.625 |
| CH32 | S14-D14 | L-MC | BA 3/4/6 (S1 / M1 / Pre-motor) | 45.309 | -24.523 | 67.388 |
| CH33 | S15-D9 | L-PFC | BA 10/11 (Frontopolar / Orbitofrontal) | 38.728 | 63.731 | -10.347 |
| CH34 | S15-D12 | L-PFC | BA 10/46 (Frontopolar / DLPFC) | 47.187 | 54.751 | 4.088 |
| CH35 | S15-D15 | L-PFC | BA 47/11 (Inferior PFC / Orbitofrontal) | 50.865 | 46.705 | -14.267 |
| CH36 | S16-D13 | L-MC | BA 9/8 (DLPFC / Frontal eye fields) | 51.185 | 22.478 | 41.803 |
| CH37 | S16-D14 | L-MC | BA 6 (Pre-motor & SMA) | 55.868 | -2.668 | 52.195 |
| CH38 | S16-D16 | L-MC | BA 6 (Pre-motor & SMA) | 64.181 | -3.74 | 36.304 |
| CH39 | S17-D14 | L-MC | BA 2/1/40 (S1 / Supramarginal gyrus) | 55.873 | -25.241 | 57.015 |
| CH40 | S17-D16 | L-MC | BA 40/2/1 (Supramarginal gyrus / S1) | 67.269 | -27.358 | 43.352 |
| CH41 | S18-D12 | L-PFC | BA 46 (DLPFC) | 53.777 | 38.39 | 17.367 |
| CH42 | S18-D15 | L-PFC | BA 47/45 (Inferior PFC / Pars triangularis) | 57.391 | 34.321 | -3.39 |

**ROI Summary:**

R-PFC (Right Prefrontal Cortex): CH1, CH2, CH8, CH9, CH10, CH17, CH18 — 7 channels

L-PFC (Left Prefrontal Cortex): CH25, CH26, CH33, CH34, CH35, CH41, CH42 — 7 channels

R-MC (Right Motor Cortex): CH3–CH7, CH11–CH16, CH19–CH21 — 14 channels

L-MC (Left Motor Cortex): CH22–CH24, CH27–CH32, CH36–CH40 — 14 channels

Total: 42 channels (14 PFC + 28 MC)

*Note: Coordinates are in MNI space, derived from the Talairach daemon registration implemented in NirSpark (Danyang Huichuang Medical Devices, China). Primary Brodmann area assignment was based on the highest probability percentage. For channels with multiple contributing BA regions, the dominant area is reported; full probability distributions are available in the dissertation appendix.*
